# An m6A-Related Prognostic Biomarker Associated With the Hepatocellular Carcinoma Immune Microenvironment

**DOI:** 10.3389/fphar.2021.707930

**Published:** 2021-06-24

**Authors:** Yingxi Du, Yarui Ma, Qing Zhu, Tongzheng Liu, Yuchen Jiao, Peng Yuan, Xiaobing Wang

**Affiliations:** ^1^ State Key Laboratory of Molecular Oncology, National Cancer Center/National Clinical Research Center for Cancer/Cancer Hospital, Chinese Academy of Medical Sciences and Peking Union Medical College, Beijing, China; ^2^ College of Pharmacy, Jinan University, Guangzhou, China; ^3^ National Cancer Center/National Clinical Research Center for Cancer/Cancer Hospital, Chinese Academy of Medical Sciences and Peking Union Medical College, Beijing, China

**Keywords:** M6A, hepatocellular carcinoma, prognosis, immune microenvironment, therapy

## Abstract

**Background:** N6-methyladenosine (m6A) is related to the progression of multiple cancers. However, the underlying influences of m6A-associated genes on the tumor immune microenvironment in hepatocellular carcinoma (HCC) remain poorly understood. Therefore, we sought to construct a survival prediction model using m6A-associated genes to clarify the molecular and immune characteristics of HCC.

**Methods:** HCC case data were downloaded from The Cancer Genome Atlas (TCGA). Then, by applying consensus clustering, we identified two distinct HCC clusters. Next, four m6A-related genes were identified to construct a prognostic model, which we validated with Gene Expression Omnibus (GEO) and International Cancer Genome Consortium (ICGC) datasets. Additionally, the molecular and immune characteristics in different subgroups were analyzed.

**Results:** m6A RNA methylation regulators were differentially expressed between HCC and normal samples and linked with immune checkpoint expression. Using consensus clustering, we divided HCC samples into two subtypes with distinct clinical features. Cluster 2 was associated with unfavorable prognosis, higher immune checkpoint expression and immune cell infiltration levels. In addition, the immune and carcinogenic signaling pathways were enriched in cluster 2. Furthermore, we constructed a risk model using four m6A-associated genes. Patients with different risk scores had distinct survival times, expression levels of immunotherapy biomarkers, TP53 mutation rates, and sensitivities to chemotherapy and targeted therapy. Similarly, the model exhibited an identical impact on overall survival in the validation cohorts.

**Conclusion:** The constructed m6A-based signature may be promising as a biomarker for prognostics and to distinguish immune characteristics in HCC.

## Introduction

Epidemiological surveys have found that hepatocellular carcinoma (HCC) is among the most lethal cancers in terms of its incidence and mortality (Bray et al., 2018). Despite great improvements in diagnosis and treatment, the overall prognosis of HCC patients remains unsatisfactory. Therefore, predictive prognostic biomarkers of HCC are immediately needed to improve the clinical outcome of HCC patients.

Recently, inhibiting immune checkpoints, such as cytotoxic T lymphocyte-associated protein 4 (CTLA4), programmed death 1 (PD1), and programmed death-ligand 1 (PD-L1), has shown clear benefits in the survival of cancer patients (Larkin et al., 2015; Bellmunt et al., 2017; Garassino et al., 2020). Compared with traditional therapies, immune checkpoint inhibitor (ICI) treatment has become an emerging strategy for HCC therapy with a significantly favorable outcome (El-Khoueiry et al., 2017; Zhu et al., 2018). Nonetheless, a major limitation is the low response rate of patients to immunotherapy (Ferris et al., 2016; Ma et al., 2019). Multiple factors, including the tumor immune microenvironment (TIME), can affect ICI effectiveness, and few biomarkers can effectively predict patient outcomes (Nishino et al., 2017). The identification of potential prognostic markers associated with treatment benefit will allow individualized immunotherapy for HCC patients. Unfortunately, we know little about the TIME of HCC. Therefore, sensitive prognostic and therapeutic biomarkers are urgently needed to predict the HCC response to ICIs. Many studies on the TIME have demonstrated the critical function of infiltrating immune cells in cancer development and the therapeutic response to immunotherapy (Jiang et al., 2018; Zeng et al., 2018). For example, tumor-infiltrating lymphocytes (TILs), such as CD4+ T cells and CD8+ T cells, have emerged as potential prognostic factors for therapeutic responsiveness to immunotherapy (Vassilakopoulou et al., 2016). Due to the high enrichment of infiltrating regulatory T cells (Tregs) and exhausted CD8+ T cells in HCC, the regulatory imbalance in the tumor immune microenvironment has an important impact on the initiation, progression and resistance of HCC, which possesses features of immunosuppressive disease (Zheng et al., 2017; Ringelhan et al., 2018; Ruf et al., 2020). Therefore, the potential mechanisms that regulate the tumor immune microenvironment should be further clarified to enable the determination of precise and accurate biomarkers that effectively render a prognosis and predict the immune response to personalized immunotherapy.

N6-methyladenosine (m6A) is the most important and prevalent internal modification of mRNA (Wang et al., 2017). m6A regulators comprise three types of factors: writers, readers, and erasers (Yang et al., 2018). A variety of studies have shown that m6A regulators make a huge difference in the modification of noncoding RNAs, including microRNAs, long non-coding RNAs, and circular RNAs, gene expression, alternative splicing, and protein translation (Dominissini et al., 2012; Alarcón et al., 2015; Lin et al., 2019; He et al., 2020; Rao et al., 2020). The aberrant expression levels of m6A regulators are tightly linked to stem cell differentiation, germ cell maturity and fertility, T cell differentiation, heart disease, and nervous system activity (Geula et al., 2015; Li et al., 2017a; Fang et al., 2020). m6A RNA methylation regulators have also been investigated extensively in a variety of tumors (Huo et al., 2020). m6A methylation has a great effect on the tumor development and progression by modulating the expression of a wide variety of oncogenes and cancer suppressor genes. For instance, depletion of METTL3 makes pancreatic cancer cells sensitive to anticancer therapy (Taketo et al., 2018). Additionally, downregulation of METTL14 may serve as a prognostic factor for HCC patients (Weng et al., 2018).

Although many efforts have been made to study the intrinsic mechanisms of m6A-related regulators in cancer progression and metastasis, the underlying roles of m6A regulators in the immune microenvironment continue to be largely unclear. A study found that neoantigen-dependent tumor-specific immunity is considerably controlled by YTHDF1 (Han et al., 2019). In addition, FTO might decrease the response to anti-PD-1 blockade immunotherapy in melanoma (Yang et al., 2019). These results suggested that m6A-associated genes might become underlying predictive factors and therapeutic targets to improve the clinical response to ICI treatment. However, whether m6A RNA methylation regulators are correlated with the TIME or immune checkpoints such as PD-L1 is unknown in HCC. There is no doubt that a comprehensive understanding of m6A-associated genes in HCC still needs to be further demonstrated.

A significant amount of research has focused on constructing a useful tool to provide better survival prediction for patients with HCC. However, little of this research has been useful. To systematically analyze the correlations of m6A-associated regulators with prognosis, the expression of immune checkpoints, therapeutic response, and TIME in HCC, we carried out this study. Specifically, we studied the expression levels of m6A-associated genes in HCC and normal tissues and the correlation between m6A regulators and immune checkpoints. Then, we identified two different HCC subtypes that had different prognostic outcomes and clinicopathological features. Next, we established a risk model for m6A-related regulators to improve the accuracy of their prognosis for HCC, which led to the categorization of HCC samples in the TCGA, GEO, and ICGC cohorts into two risk subgroups. Next, the relationships between the risk models and immune checkpoints, immune cell infiltration levels, total mutation burden (TMB), neoantigen counts, gene mutation status, and therapeutic sensitivity were fully elucidated on the basis of the m6A-related signature to systematically examine the effects of m6A regulators on the survival and tumor immune microenvironment of HCC. These results demonstrated that m6A-associated regulators play key roles in HCC prognosis, TIME, and therapeutic responses.

## Methods

### Data Collection

The mRNA expression profiles and the corresponding clinicopathological data of HCC patients were simultaneously downloaded from the TCGA on August 3, 2020, and consisted of data on 374 HCC and 50 normal case samples. The RNA-seq data and survival data of 221 HCC samples (GSE14520 dataset) were obtained from the GEO database. The RNA-seq data and clinical information of another 232 HCC cases were downloaded from the ICGC database. We removed the batch effect *via* the “sva” R package.

### Identification of m6A RNA Methylation Regulators

After searching the recently published literature on m6A, we found 18 m6A-related genes (Huo et al., 2020). A total of 15 of these genes were selected based on the mRNA expression data of HCC obtained from the TCGA. Ten genes (YTHDC2, FTO, ZC3H13, YTHDC1, YTHDF3, YTHDF1, METTL3, RBM15, YTHDF2, and WTAP) were identified for subsequent prognostic analysis because they are listed in the GSE14520 dataset.

### Construction and Validation of the Prognostic Gene Signature

The risk model of four m6A regulators was constructed using least absolute shrinkage and selection operator (LASSO) regression analysis of the TCGA cohort data. The coefficients were derived from the LASSO regression analysis. The risk score was obtained from the equation: risk score = $\overset{n}{\underset{i=1}{\sum }}\left(\text{coefficient of mRNAi*expression of mRNAi}\right)$. Then, according to the median value of the risk score, HCC patients were classified into the high-risk subgroup or the low-risk subgroup.

### Gene Ontology and Gene Set Enrichment Analysis

To elucidate the biological features of two distinct clusters, the “clusterProfiler” package was employed for the Gene Ontology (GO) enrichment analysis, and *p*. adjust <0.05 showed significance (Yu et al., 2012). GSEA was carried out using the Hallmark gene set “h.all.v7.0.symbols.gmt” to illustrate the different enriched terms between different HCC subtypes.

### Immune Cell Infiltration Estimation, Tumor Mutation Burden and Neoantigen Analyses

The immune score and stromal score for each patient were obtained by using the “estimate” package (Yoshihara et al., 2013). The immune cell infiltration levels were assessed comprehensively through The Tumor Immune Estimation Resource (TIMER), which estimates the abundance of six immune cell infiltrates (Li et al., 2017b). The somatic mutational profile of HCC was downloaded from the TCGA. The quantity and quality of the gene mutations were analyzed in the two groups with the “Maftools” package of R (Mayakonda et al., 2018). Neoantigens in the TCGA-LIHC dataset were obtained from a previously published study (Thorsson et al., 2018).

### Chemotherapy and Targeted Therapy Response Prediction

To estimate the predictive role of the model for HCC treatment, we used the R package “pRRophetic” to evaluate the half maximal inhibitory concentration (IC50) of common chemotherapy and targeted therapy drugs, such as sorafenib, mitomycin, and doxorubicin.

### Statistical Analysis

Statistical tests were conducted using R version 4.0.2 and GraphPad Prism 8.0. The group comparisons of two groups were compared by *t*-test. The expression correlation analysis between m6A-associated genes and immune checkpoints was performed by Pearson correlation test. Using “km” method in the R package “ConsensusClusterPlus,” we classified 374 HCC patients into different subtypes. We performed a chi-square test to explore the relationship between the clusters and clinicopathological characteristics. Survival curves and survival differences were generated by the Kaplan-Meier method with log rank test. Univariate and multivariate analyses were performed by adopting the Cox regression method to determine whether the risk score combined with other clinical characteristics was an independent prognostic factor. In addition, a receiver operating characteristic (ROC) curve analysis was performed to evaluate the predictive power of the prognostic model. A *p* value (two-sided) less than 0.05 was accepted as statistically significant: not significant (ns), *p* < 0.05 (*), *p* < 0.01 (**), *p* < 0.001 (***) and *p* < 0.0001 (****).

## Results

### m6A RNA Methylation Regulators Were Largely Overexpressed and Associated With Immune Checkpoints in Hepatocellular Carcinoma

**FIGURE 1:**
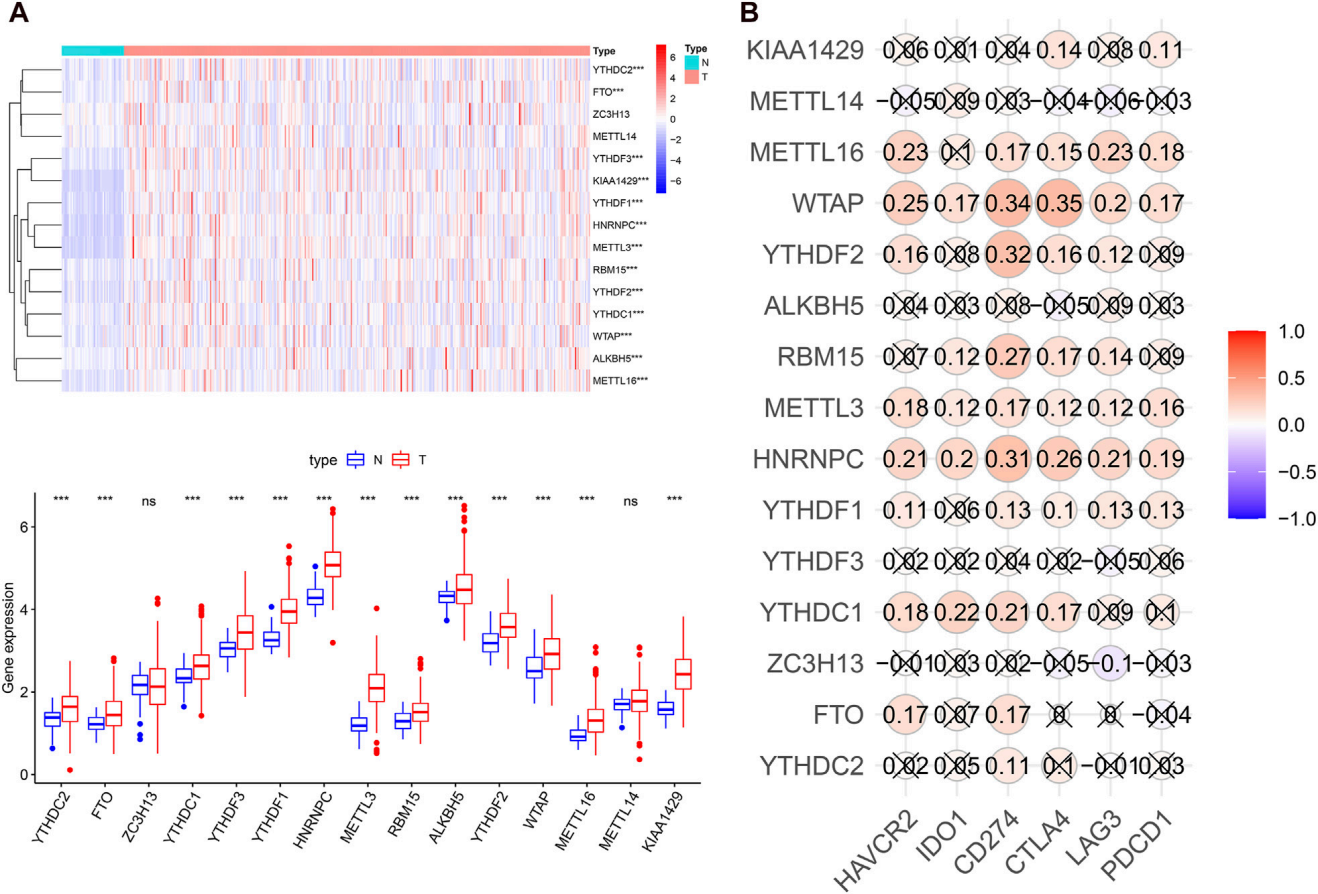
m6A regulators expression levels and correlation with immune checkpoint expression in HCC. **(A)**, heatmap and boxplot of 15 m6A-related genes expression in HCC. N: normal tissue; T: tumor tissue. **(B)**, the expression correlation between the immune checkpoints and m6A regulators. “X” means *p* > 0.05.

To systematically investigate the potential impact of m6A-related genes on HCC development and progression, we assessed the distinct expression levels of 15 m6A-associated genes between HCC and normal tissues in the TCGA dataset. It was evident that the expression levels of m6A-associated genes in the HCC and normal patients were different (Figure 1A). All m6A regulators, except METTL14 and ZC3H13, were significantly overexpressed in the HCC samples. Then, we assessed the correlation of immune checkpoints, including HAVCR2 (also known as TIM3), LAG3, PD-L1, CTLA4, IDO1, and PD1, with m6A-related genes. The expression levels of immune checkpoints showed a positive correlation with m6A-associated genes (Figure 1B). These findings demonstrated that m6A-related regulators might have essential effects on HCC development and progression. Considering the known roles of checkpoints in the immunosuppressive microenvironment, m6A-associated regulators may have crucial biological functions in HCC immunotherapy.

### The Association of Consensus Clustering With the Clinicopathological Features, Survival Status, Tumor Signaling Pathways, and Immune Cell Infiltration in Hepatocellular Carcinoma

**FIGURE 2:**
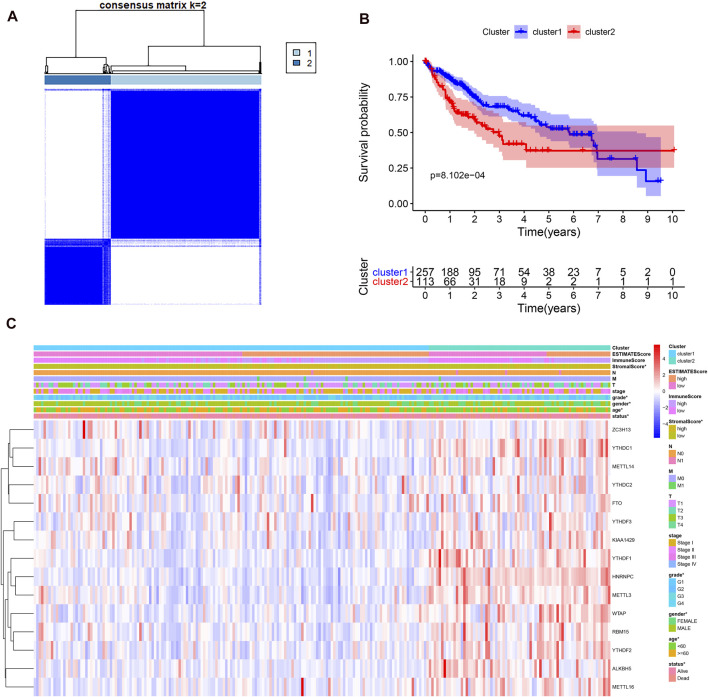
Different clinicopathological characteristics and survival of HCC in two clusters in the TCGA-LIHC. **(A)**, consensus clustering heatmap for k = 2 in HCC. **(B)**, survival curve of overall survival in two clusters. **(C)**, heatmap and clinicopathological characteristics of the two clusters.

**FIGURE 3:**
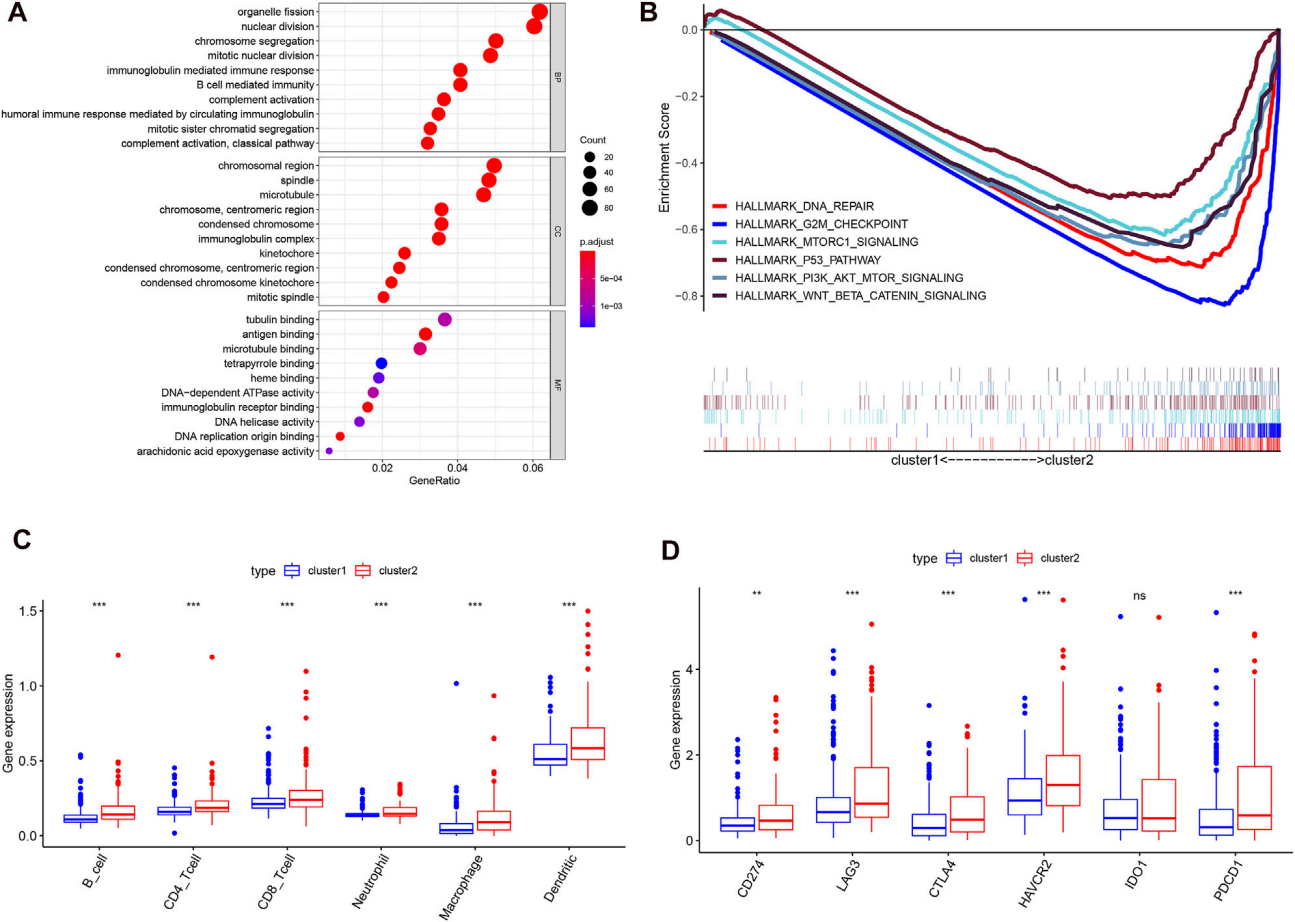
Different cancer pathways and immune cell infiltration level in two subtypes in the TCGA-LIHC. **(A)**, top enriched pathways in distinct two clusters. **(B)**, several differential enriched pathways in GSEA. **(C)**, the infiltrating level of six immune cell types in two clusters. **(D)**, the expression level of immune checkpoints in two clusters.

Considering the optimal clustering stability, k = 2 was ultimately identified (Supplementary Figure S1), and the samples from 374 patient with HCC were categorized into two different subtypes (Figure 2A). Then, the overall survival (OS) and other clinical information of cluster 1 (*n* = 260) and cluster 2 (*n* = 114) were compared comprehensively. The OS (*p* = 0.0008) of cluster 1 with downregulated m6A regulator expression was better than that of cluster 2 with upregulated m6A regulator expression (Figure 2B). We found that the expression level of individual m6A-associated genes in cluster 2 was higher than that in cluster 1, expect that of ZC3H13. Then, we fully compared the clinical characteristics between the two clusters (Figure 2C). Cluster 1 mostly consisted of samples from male and elderly patient with HCC (*p* < 0.05). Cluster 2 was closely linked with a higher histological grade and a lower stromal score than cluster 1 (*p* < 0.05). We also performed PCA to find the gene expression profiles that differed between the two subtypes (Supplementary Figure S1D). The results indicated that the clusters defined by m6A-related genes were tightly linked to HCC tumor heterogeneity. Next, to further explore potential functional pathways, Gene Ontology (GO) enrichment analysis was conducted for the differentially expressed genes (DEGs) between the two subtypes. The DEGs were closely related to biological processes (BPs) of the immune response, such as the immunoglobulin-mediated immune response and complement activation (Figure 3A). Because of the possible difference in the immune microenvironment between the two clusters, GSEA was conducted to further analyze the underlying regulatory mechanisms. The findings revealed that cluster 2 samples expectedly possessed several canonical hallmarks of malignancy, such as DNA repair, G2M checkpoint, mTORC1 pathway, Wnt/β-Catenin pathway, P53 pathway, and PI3K/AKT/mTOR pathway (Figure 3B). Hence, these pathways linked with the development and progression of cancers, particularly the PI3K/AKT/mTOR pathway, P53 pathway, and Wnt/β-Catenin pathway, might be connected with the distinct immune microenvironment of the two clusters. To explore the influence of m6A-associated regulators on the tumor immune microenvironment of HCC, we assessed the immune cell infiltrate level, immune score, and stromal score between the two clusters. The stromal score was significantly different between the two clusters (Figure 2C). Next, the infiltration levels of immune cells in the two clusters were explored. Cluster 2 samples showed a higher abundance of immune cells (Figure 3C). To further investigate the involvement of immune checkpoints with m6A-associated genes, we evaluated the expression levels of immune checkpoints between the two clusters. The immune checkpoints were highly expressed in cluster 2 samples (*p* < 0.01; Figure 3D). The expression levels of immune checkpoints were also compared between HCC and normal patient samples (Supplementary Figure S2A). Given these results, the patients represented by cluster 2 may potentially have a higher response rate to ICI treatment.

### Construction of an m6A RNA Methylation Regulator-Based Prognostic Model

**FIGURE 4:**
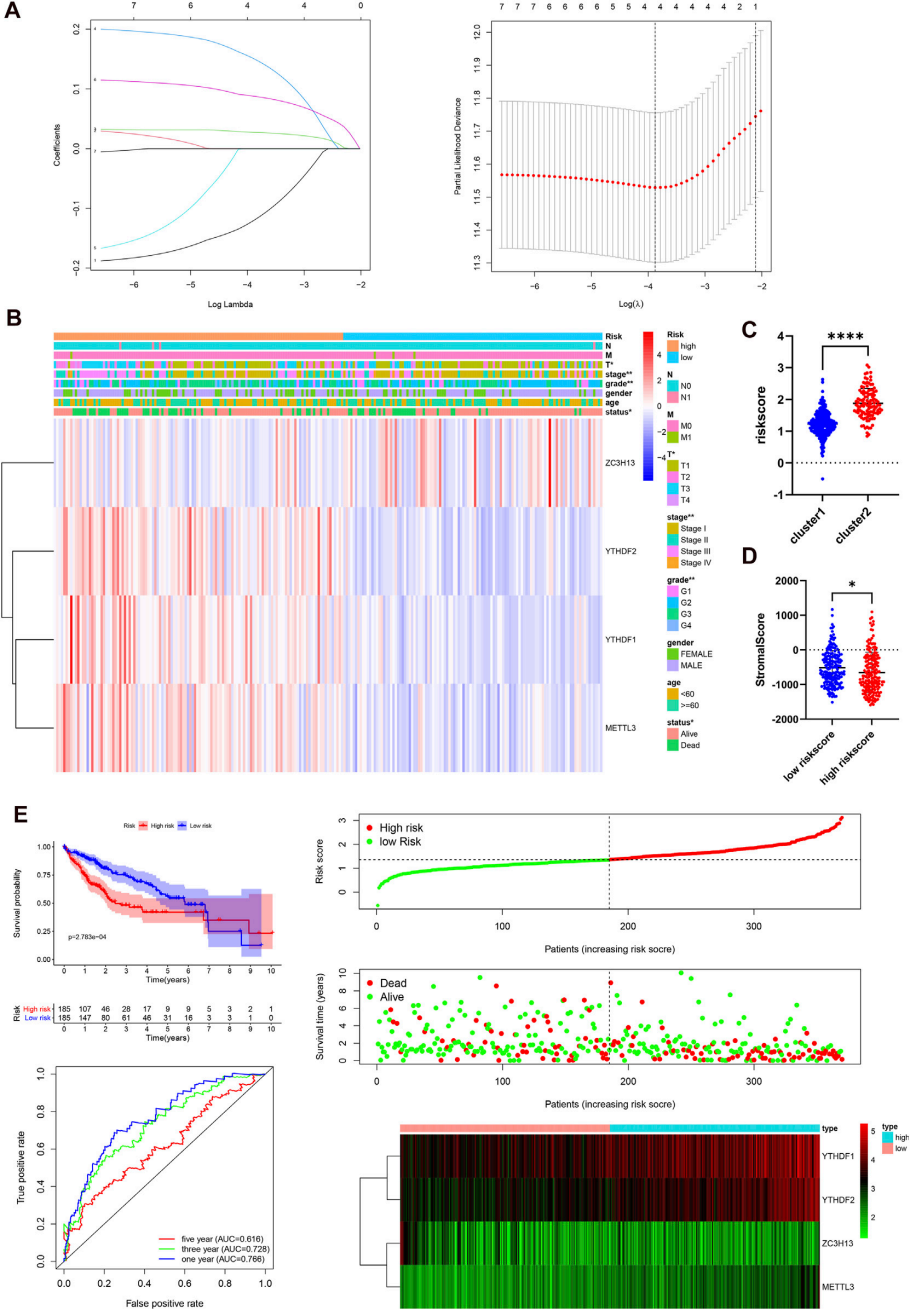
Prognostic risk signatures correlated with clinicopathological characteristics in the TCGA-LIHC. **(A)**, construction of the risk signature model. **(B)**, heatmap and clinicopathological characteristics of two risk subgroups. **(C)**, distribution of risk scores stratified by cluster. **(D)**, the relationship between risk score and stromal score. **(E)**, survival curve, ROC curve, and risk score analysis in the TCGA-LIHC cohort.

Next, we elucidated the prognostic function of m6A-related genes in HCC patients. Univariate Cox regression analysis was used to identify seven survival-related genes (Supplementary Figure S2B). Then, LASSO regression analysis was performed based on the expression levels of seven identified m6A-associated genes in the TCGA cohort. As a consequence, four m6A-related genes, namely, METTL3, YTHDF2, YTHDF1, and ZC3H13, were identified (Figure 4A). The risk score of each patient in the TCGA, GEO, and ICGC datasets was calculated by employing the following equation: risk score = (0.1503* expression level of METTL3) + (0.0877* expression level of YTHDF2) + (0.0274* expression level of YTHDF1) − (0.1197* expression level of ZC3H13). Subsequently, 185 cases were classified into the high-risk group and 185 cases were classified into the low-risk group according to the median risk score in the TCGA dataset. We studied the associations between the risk score and clinicopathological characteristics. The heatmap results showed that the four m6A regulators had distinct expression levels in the two risk subgroups in the TCGA cohort (Figure 4B). METTL3, YTHDF2, and YTHDF1 were mainly overexpressed in the high-risk subgroup, whereas ZC3H13 was upregulated in the low-risk subgroup. The differences in status (*p* < 0.05), grade (*p* < 0.01), stage (*p* < 0.01), and T stage (*p* < 0.05) between the two risk subgroups were significant. In addition, we studied the associations between risk score and clustering subtypes and stromal score. Not surprisingly, we found that patients in cluster 2 showed an evidently higher risk score than patients in cluster 1 (*p* < 0.0001, Figure 4C). Compared to the group of patients with a high-risk score, the group of patients with a high-risk score had a higher stromal score (*p* < 0.05, Figure 4D). The immune score between two groups was no statistical significance (Supplementary Figure S2C). To further test the robustness of the risk model, we plotted a Kaplan-Meier curve. Patients in the high-risk group had a reduced survival time compared with those in the low-risk group (*p* < 0.001). In addition, the time-dependent ROC analysis were performed to assess the predictive accuracy of the risk model, and the area under the curve (AUC) was as high as 0.766 at 1 year, 0.728 at 3 years, and 0.616 at 5 years (Figure 4E). These findings indicate that the risk score was dramatically related to clustering subtypes, degree of liver cancer malignancy, stromal score, and survival time for patients with HCC.

### The Independent Prognostic Role of the Risk Model Based on the Gene Expression Omnibus and International Cancer Genome Consortium Cohorts

**FIGURE 5:**
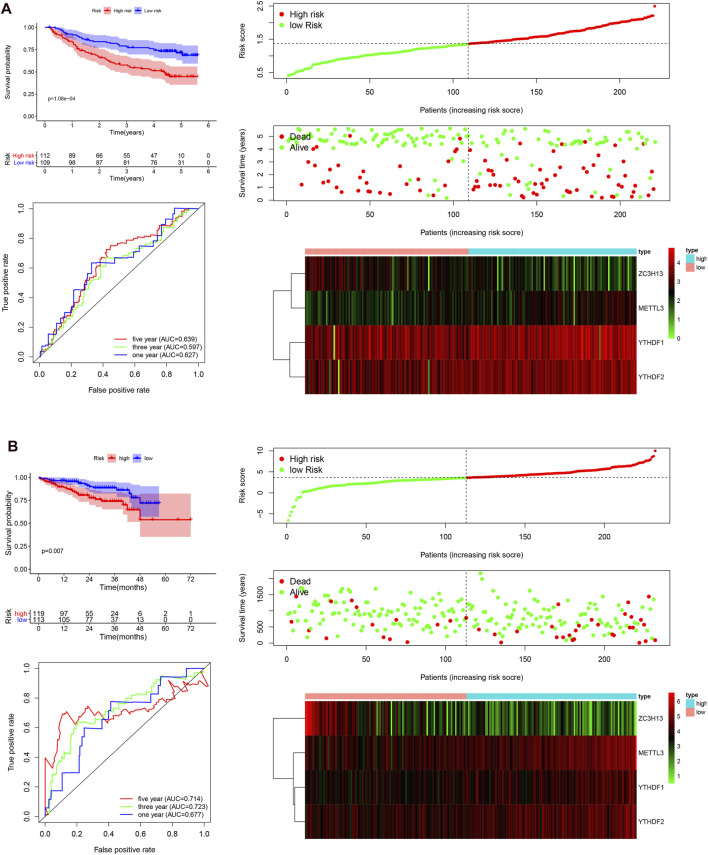
Survival curve analysis, time-dependent ROC curve analysis, and risk score analysis in the GEO and ICGC datasets. **(A)**, survival curve, ROC curve, and risk score analysis in the GSE14520 cohort. **(B)**, survival curve, ROC curve, and risk score analysis in the ICGC cohort.

To validate that the m6A-associated genes had a similar impact on other HCC cases, we selected the GSE14520 dataset and ICGC dataset to serve as the external validation cohorts. Patient data were then categorized into two groups according to the median risk score of the TCGA cohort. The overall survival status, ROC curves, and expression details related four selected m6A-related genes and the corresponding risk scores in the GEO and ICGC cohorts are exhibited in Figure 5. The heatmaps show that METTL3, YTHDF2, and YTHDF1 are mainly overexpressed in the high-risk subgroup, whereas ZC3H13 is upregulated in the low-risk subgroup, serving as a protective m6A regulator. The 1, 3, and 5 years ROC curves in this model showed that the AUC values were moderate. The patients in the low-risk group exhibited a longer survival time than those in the high-risk group (*p* < 0.001; Figure 5A). Similarly, in the ICGC cohort, the patients in the high-risk subgroup showed a shorter survival time (*p* = 0.007; Figure 5B), and the 1, 3, and 5 years AUC values were also moderate. Hence, the AUC values demonstrated that the four risk signatures were effective for distinguishing HCC patient outcomes. Taken together, our findings demonstrate that the risk score that was obtained based on the four m6A-associated genes might have a high accuracy and precision for predicting the clinical outcome of HCC patients. Next, univariate and multivariate Cox regression analyses were carried out with the TCGA, GEO, and ICGC datasets (Supplementary Figure S3). All analyses demonstrated that the risk score was closely linked with the survival time of the patients with information in the TCGA, GEO, and ICGC datasets. The findings confirmed that the risk score based on the four m6A regulators serve as independent prognostic factors in HCC patients.

### The Mutational Landscape and Therapeutic Sensitivity of Different Subgroups

**FIGURE 6:**
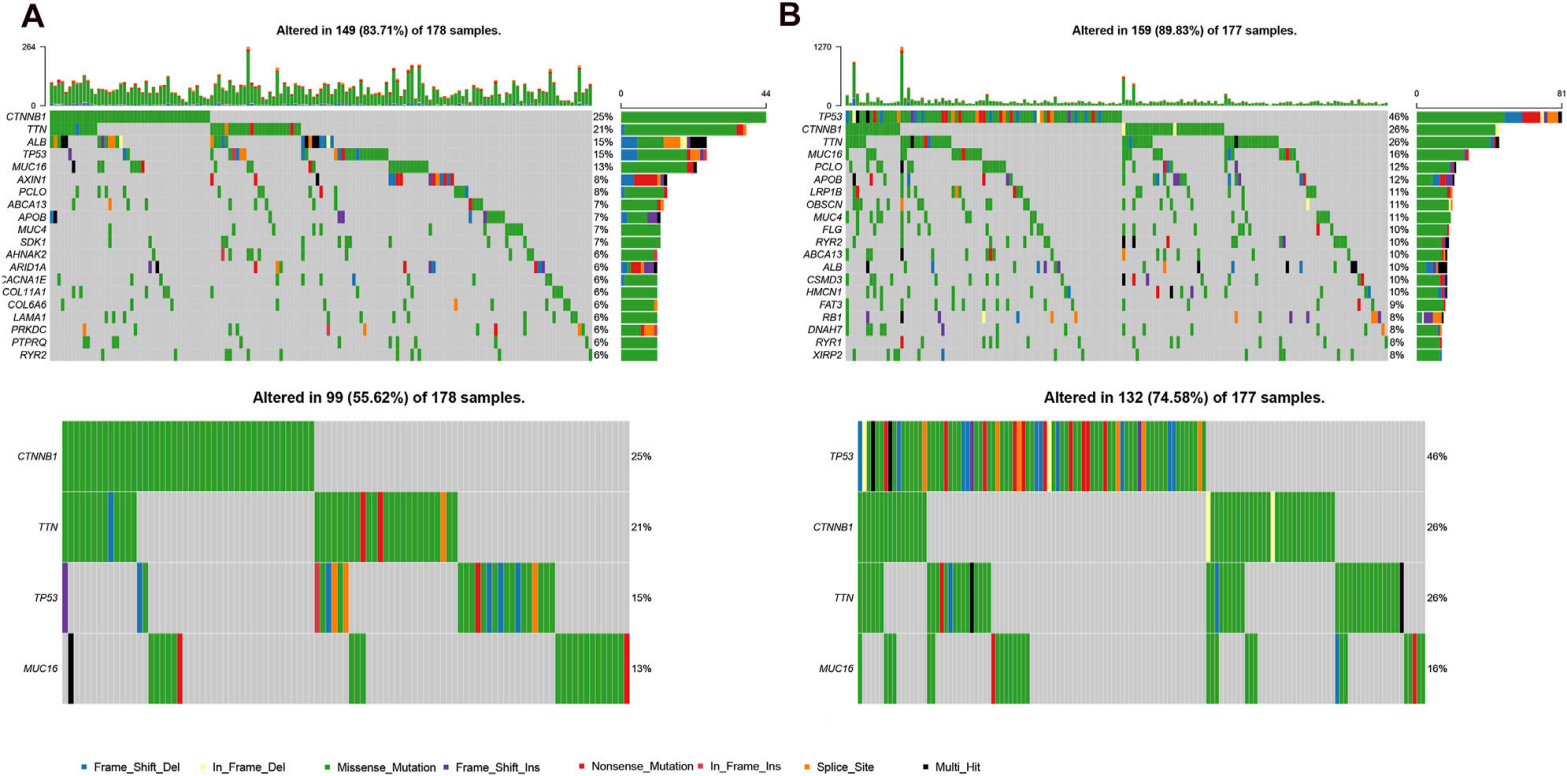
Mutational landscape of two risk subgroups in the TCGA-LIHC. **(A,B)**, genes with high frequency mutation in the HCC samples of low-risk subgroup **(A)** and high-risk subgroup **(B)**.

**FIGURE 7:**
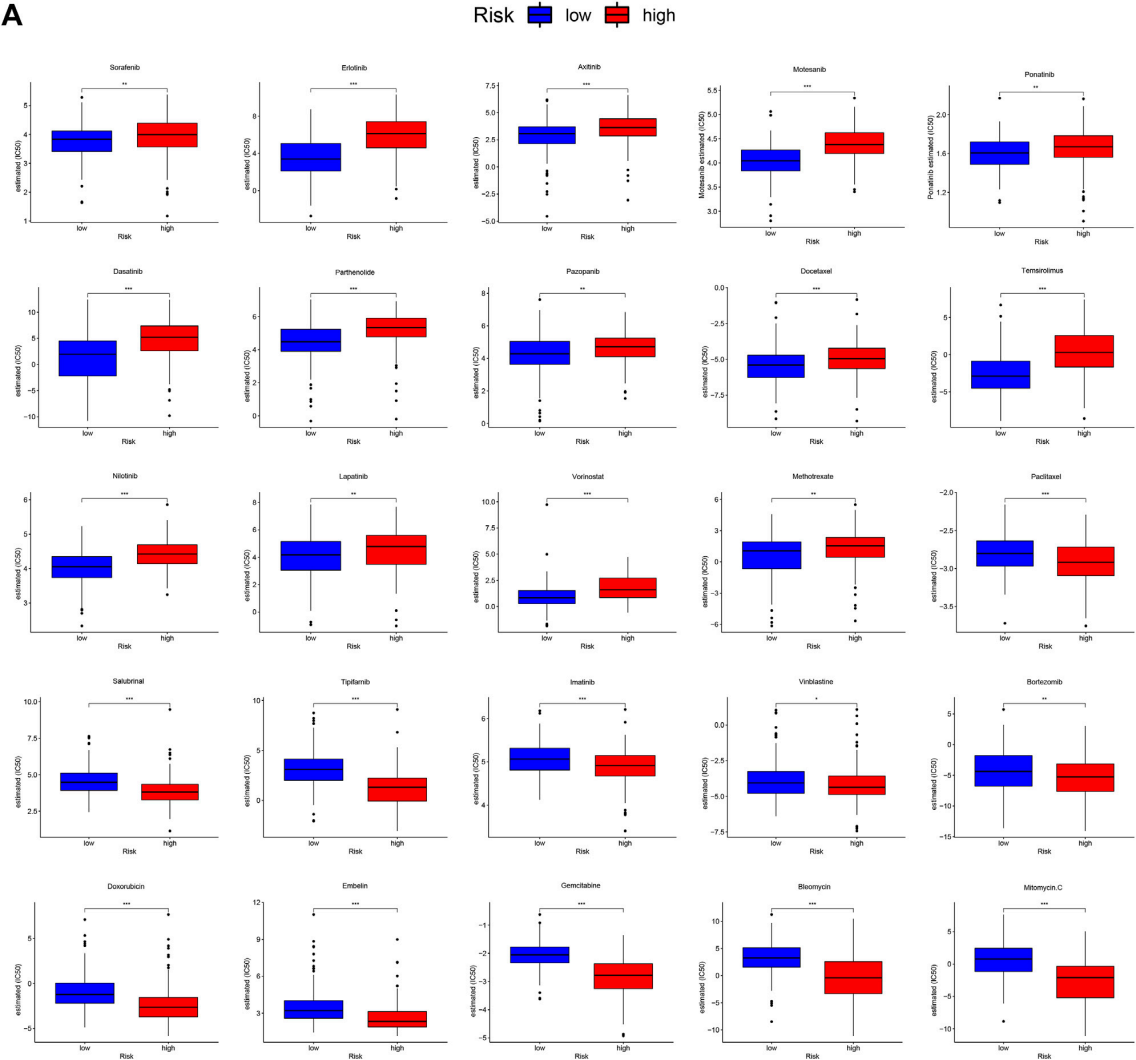
Differential chemotherapy and targeted therapy responses in two risk groups. **(A)**, the high-risk score was related to a higher IC50 for chemotherapy and targeted therapy such as sorafenib, whereas it was related to a lower IC50 for drugs such as mitomycin and doxorubicin.

Because gene mutation status has been shown to impact the survival time of patients with HCC, we assessed the distribution of somatic variants in HCC driver genes between the two subgroups (Supplementary Figure S4A). The analysis demonstrated that missense variations were the most frequent mutation type in HCC. As shown in Figure 6, we then identified the top 20 genes with the highest mutation rates in the two risk subgroups. The mutation rates of TP53, CTNNB1, MUC16, ALB, and TTN were higher than 10% in both groups. Mutation of the TP53 gene was more common in the high-risk subgroup, while mutation of the CTNNB1 gene was the most common in the low-risk subgroup. These results might provide innovative insights for elucidating the distinct mechanisms of tumor progression. Genetic mutations can affect the tumor response to chemotherapy and targeted therapy; therefore, we investigated the association between the risk model and the efficacy of chemotherapy and targeted therapy drugs in patients with HCC. As shown in Figure 7, we listed 25 common drugs used for HCC, such as sorafenib, mitomycin, and doxorubicin. Significant differences in the estimated IC50 between the two risk groups were observed, which suggest that the risk model might be used to identify potential biomarkers for chemotherapy and targeted therapy sensitivity. Then, we tested the ability of the signature to predict the efficacy of sorafenib treatment in TCGA cohort (Supplementary Figures S4B,4C). We found that a weak tendency for progressive disease (PD) patients and high-risk patients to have a poorer OS was observed. Surprisingly, we found that low-risk patients showed a higher response rate to sorafenib compared with high-risk patients.

### The Correlation Between the Risk Score and Immune Characteristics in Hepatocellular Carcinoma

**FIGURE 8:**
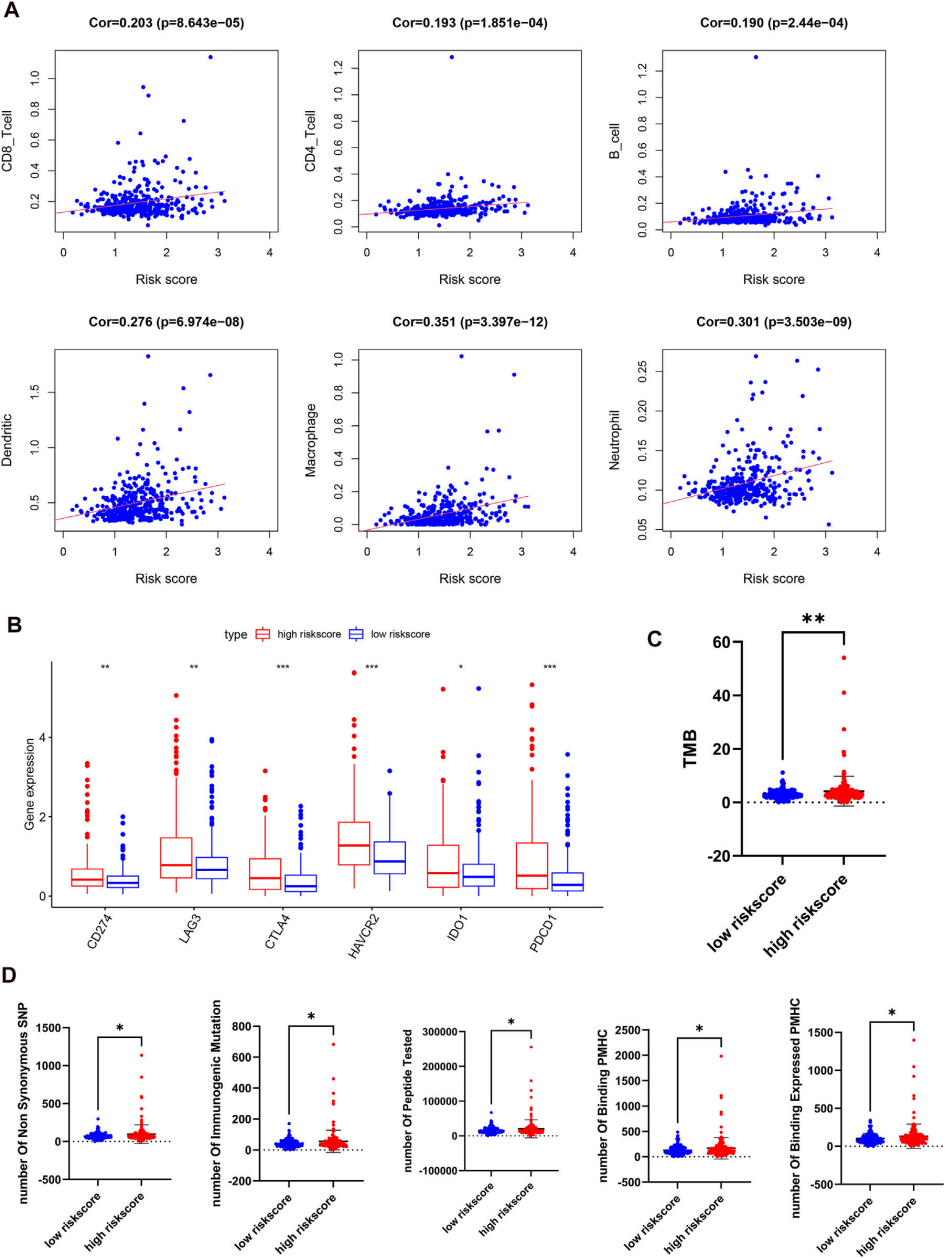
Distant immune features in two risk subgroups in the TCGA-LIHC. **(A)**, the correlation between the risk score and the immune cell infiltration. **(B)**, the expression levels of immune checkpoints in two risk subgroups. **(C,D)**, tumor mutation burden **(C)** and neoantigen **(D)** were compared with the two risk subgroups.

Because of the relationship between m6A regulators and immune-related biological pathways, the impact of the risk model on the TIME in HCC was investigated. The risk score had a positive correlation with the infiltration levels of 6 immune cell types (*p* < 0.001, Figure 8A). This finding suggested that the risk score is intimately involved in the TIME for patients with HCC. Then, the expression levels of immune checkpoints such as PD-L1, IDO1, PD1, LAG3, CTLA4, and TIM3 between the two risk groups were examined. It is obvious that the high-risk subgroup overexpressed PD-L1, LAG3, IDO1, PD1, CTLA4, and TIM3 (Figure 8B). Next, we studied the relationships between the risk score and total mutation burden (TMB), and neoantigens. We found that the TMB and neoantigen counts in the high-risk subgroup were very high (Figures 8C,D). Our findings revealed that the risk score is related to vital regulatory functions in the immune microenvironment in HCC and may indicate the extent of a tumor response to immunotherapy, especially ICI treatment.

### Genetic Alterations and Expression Levels of Four Predictive m6A-Related Regulators in Hepatocellular Carcinoma

**FIGURE 9:**
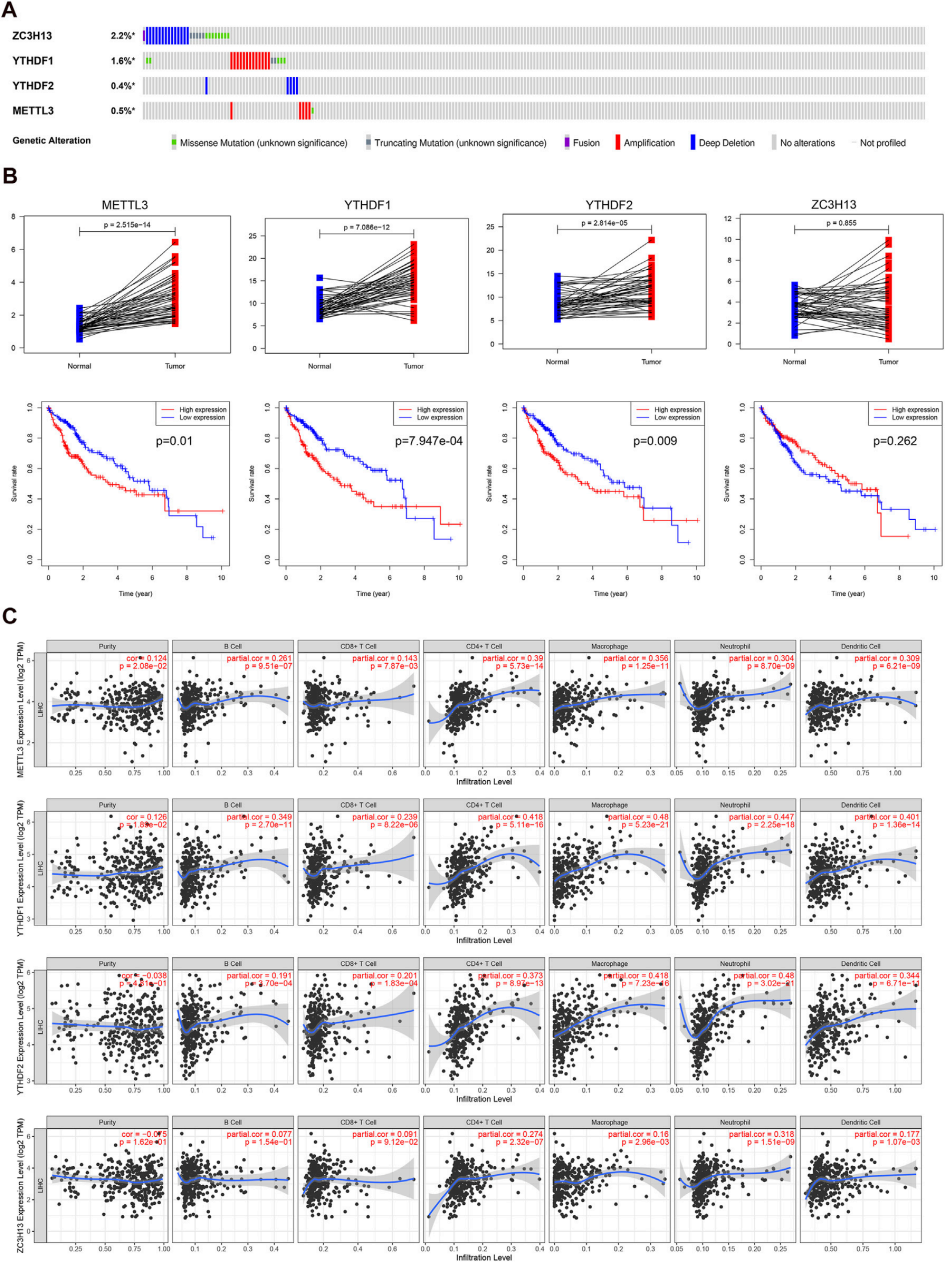
Genetic alterations, expression levels and prognosis, and correlation with immune cells of the four predictive genes. **(A)**, genetic alterations of the four m6A-associated regulators in the TCGA cohort. **(B)**, the expression levels and survival outcomes of the four genes in the TCGA cohort. **(C)**, effects of the expression levels of four m6A-associated regulators on the immune cell infiltration.

Finally, we analyzed the genetic alterations, expression levels, overall survival, and infiltration levels of immune cells of the four identified genes. We found that ZC3H13 had the most frequent genetic alterations (2.2%) among these four genes in HCC. Furthermore, deep deletion and amplification mutation were the most common alterations among these four genes (Figure 9A). Then, we compared the expression levels and prognosis of the METTL3, YTHDF1, YTHDF2, and ZC3H13 genes (Figure 9B). In accordance with our results, METTL3, YTHDF2, and YTHDF1 were found to be considerably overexpressed between tumor and normal adjacent tissues. Moreover, patients with high expression of these three genes exhibited shorter survival times. The effects of the four m6A-related genes on immune cell infiltration were further explored. We discovered that the expression levels of the identified m6A-associated regulators had a great effect on the infiltration levels of the six immune cells in HCC (Figure 9C). Our findings suggested that the identified m6A-associated regulators crucially affected the survival time and tumor immune microenvironment of patients with HCC.

## Discussion

As one of the most primary and common forms of mRNA modification, N6-methyladenosine has a tremendous effect on posttranscriptional regulation (He, 2010). Numerous studies have confirmed that the dysregulation of m6A methylation regulatory proteins is associated with the development and progression of many tumors (Pan et al., 2018). However, the functions of specific m6A regulators that serve as oncogenes or tumor suppressor genes in different tumor types are overwhelmingly complicated. For instance, several studies found that ALKBH5, a demethylase, plays distinct roles in different tumor types (Kwok et al., 2017; Zhang et al., 2017). Since most studies have made efforts to elucidate how m6A regulators modulate intrinsic tumor carcinogenic signaling pathways, further research focusing on the potential regulatory mechanisms of m6A-associated regulators in the TIME and immune response of HCC is urgently needed.

Specifically, our results found that the expression levels of m6A-associated genes, except ZC3H13 and METTL14, were strikingly overexpressed in HCC cases compared with normal cases. Our study also showed unexpected associations between m6A regulators and immune checkpoints. Next, we identified two different HCC subtypes by consensus clustering. The two cluster subtypes had different prognostic outcomes and clinicopathological features. In addition, the two clusters were also related to the different expression of immune checkpoints and immune cell infiltration levels, which means that there was a significant difference in the TIME between the two clusters. The differences in the tumor immune microenvironment between the two clusters may have contributed to the difference in survival times, with the TIME in cluster 2 characterized by immunosuppressive cells and factors. The immune checkpoint expression levels were highly significant in cluster 2 with respect to cluster 1. That result indicates that immunotherapy such as ICI treatment may function in patients with data in cluster 2. Further analysis demonstrated that the proportions of B cells, CD4+ T cells, CD8+ T cells, neutrophils, macrophages, and dendritic cells in cluster 2 were greatly increased compared with those in cluster 1. The GO analysis suggested that immune-related pathways were mainly enriched in the cluster 2 samples. In addition, we conducted GSEA and found that the functional regulatory pathways of malignant tumors, such as the P53 pathway, Wnt/β-Catenin pathway, and PI3K/AKT/mTOR pathway, were evidently enriched in the cluster 2 samples. A previous study found that depleting m6A methyltransferase had an evident impact on gene expression, leading to the regulation of the p53 signaling pathway (Dominissini et al., 2012). In addition, the expression of p53 is regulated posttranscriptionally by m6A RNA methylation (Ghazi et al., 2020). There is also experimental evidence indicating that mutated YTHDF1 results in the m6A-mediated activation of Wnt/β-Catenin signaling and gastric carcinogenesis (Pi et al., 2020). Additionally, METTL3 exerts an angiogenic role by regulating Wnt signaling (Yao et al., 2020). m6A regulators regulate the AKT signaling activity to promote the development and progression of endometrial cancer (Liu et al., 2018). Similarly, the METTL3 expression level is associated PI3K/AKT/mTOR pathway molecule expression levels and is related to unfavorable outcomes in renal cell carcinoma (Li et al., 2017c). These results suggest that the PI3K/AKT/mTOR pathway, Wnt/β-Catenin pathway, and P53 pathway may act as potential targets for m6A-modified RNA. Therefore, the m6A modification and the P53 pathway, Wnt/β-Catenin pathway, and PI3K/AKT/mTOR pathway may be collectively associated with the modulation of the tumor microenvironment and immune response in different HCC clusters. Next, the prognostic value of the m6A-associated genes in HCC patients was assessed based on four regulators (ZC3H13, METTL3, YTHDF1, and YTHDF2). Among these four m6A-associated genes, METTL3 promotes the development and progression of HCC (Chen et al., 2018), whereas it exerts a tumor-suppressive function in breast cancer (Wang et al., 2017). ZC3H13 works as a cancer inhibitory factor in colorectal cancer (Zhu et al., 2019), but another study indicated that ZC3H13 functions as an oncogene in several types of cancers (Panahi et al., 2016). A study also demonstrated the oncogenic role of YTHDF1 in HCC (Liu et al., 2020a). YTHDF2 can promote liver cancer metastasis (Zhang et al., 2020). Importantly, the model revealed valid prognostic biomarkers for HCC. The risk score derived from four identified m6A-related genes effectively enabled the categorization of the patients with HCC into two subgroups. As expected, cluster 2 had an evidently higher risk score than cluster 1. In addition, compared with the low-risk patients, the high-risk patients in the TCGA dataset had worse overall survival. We also obtained consistent results in the GSE14520 and ICGC external datasets. Univariate and multivariate Cox regression analyses suggested that the prognostic risk model was independent of other clinical factors in HCC. In summary, this m6A regulator-associated risk model can precisely evaluate HCC patient outcomes.

The tumor immune microenvironment, which is regulated by various immune factors, has a critical effect on tumor development and progression. In addition, its dysregulation can result in multiple outcomes, such as different prognosis results and therapeutic responses to immunotherapy (Fridman et al., 2012; Hui and Chen, 2015). As an immunosuppressive disease, HCC consists of a variety of immunocompetent cells and immunosuppressive cells, including DCs, CD4+ T cells, CD8+ T cells, Tregs, and macrophages. However, the effects of m6A-related genes on the TIME in HCC still need to be understood. This study demonstrated that the risk score calculated by the four risk signatures of m6A regulators was evidently connected with the expression levels of immune checkpoints and immune cell infiltration. The risk score was significantly positively correlated with the abundance of B cells, CD4+ T cells, CD8+ T cells, neutrophils, macrophages, and dendritic cells. There have been a variety of studies focusing on the relationship of m6A regulators and the immune system. For example, a study revealed that METTL3 is closely related to homeostasis and differentiation disorders of T cells (Li et al., 2017a). Similarly, it has been found that both METTL3 and METTL14 may regulate immune responses to immunotherapy (Wang et al., 2020). Another study revealed that YTHDF1 has a negative correlation with the proportion of CD8+ T cells (Han et al., 2019). In addition, FTO plays a crucial role in promoting melanoma anti-PD-1 resistance (Yang et al., 2019). Therefore, these findings indicated that m6A-related genes are more or less associated with the dysregulation of the TIME.

To gain further biological insight into different aspects of the two subgroups, we studied gene mutations, therapeutic sensitivity, TMB, and neoantigens of different subgroups. The largest difference in mutations between these two groups was TP53 mutation, which was more common in the high-risk samples than in the low-risk samples (46% vs. 15%). TP53 mutation is not only the most common single genetic variation in cancer but is also associated with additional unfavorable outcomes in various cancers, particularly HCC (Kandoth et al., 2013; Muller and Vousden, 2014). In contrast, the CTNNB1 mutation was the most frequent mutation in the low-risk subgroup, which may indicate that low-risk HCCs promote proliferation through the Wnt/β-Catenin signaling pathway (Delgado et al., 2015). Therefore, high-risk patients with more TP53 mutations have a worse outcome than low-risk patients with fewer TP53 mutations. Currently, because chemotherapy and targeted therapy are common methods used to treat HCC, we found that there was a significantly different sensitivity between the two risk groups, allowing us to promote a deep understanding of personalized treatments. For example, low-risk HCC patients were more sensitive to sorafenib than high-risk HCC patients. Next, we examined the relationship between this risk score and known predictive biomarkers for immunotherapy, such as TMB and neoantigens. Here, our results revealed that the risk score had an evidently positive correlation with TMB and neoantigens, which indicated that high-risk patients may have an improved response to ICI treatment. Recently, TMB has been assessed as a promising and potential biomarker for predicting the response to ICI therapy in many clinical trials and across different tumor types, including HCC (Samstein et al., 2019). In addition, patients with higher neoantigen loads tend to show a better response to ICI therapy (Liu et al., 2020b). These results indicate that patients with HCC and high-risk scores might profit the most from immunotherapy. In conclusion, because there are significant differences in the tumor immune microenvironment and molecular characteristics, HCC patients with different risk scores may have different survival statuses and experience distinct outcomes from immunotherapy, chemotherapy, and targeted therapy.

It is undeniable that our study has some limitations. First, the proposed risk model, which was derived from four m6A regulators, was only substantiated in the TCGA, GSE14520 and ICGC cohorts. Therefore, further external validation in other external cohorts with sufficiently available information is warranted to test the accuracy and precision of this risk score model. In addition, due to the lack of specific HCC cohorts with ICI treatment, we did not evaluate the correlation between the risk model and the response to immunotherapy. Last, the interactions and regulatory mechanisms of m6A RNA methylation regulators in the tumor immune microenvironment need to be further studied to remodel the tumor immune microenvironment and enhance the efficacy of immunotherapy in HCC. Overall, in our study, we performed a systematic evaluation of the underlying regulatory mechanisms of m6A-related genes and the effects of these genes on prognosis, the expression of immune checkpoints, the infiltration of several major immune cells, the levels of TMB and neoantigens, the gene mutation rate, and therapeutic sensitivity in HCC. The risk model, which has the ability to distinguish immune and molecular characteristics, might become a helpful prognostic indicator of immunotherapy, but further studies are needed to confirm its effectiveness.

## Data Availability

The raw data supporting the conclusion of this article will be made available by the authors, without undue reservation.
