# Supplementary material for: An m6A-Related Prognostic Biomarker Associated With the Hepatocellular Carcinoma Immune Microenvironment
Source: Front Pharmacol. 2021 Jun 24;12:707930. doi: 10.3389/fphar.2021.707930 (PMC8263919; doi:10.3389/fphar.2021.707930)
Supplement: Supplementary file 1 [file DataSheet1.pdf]

Supplementary Material

Supplementary Figures

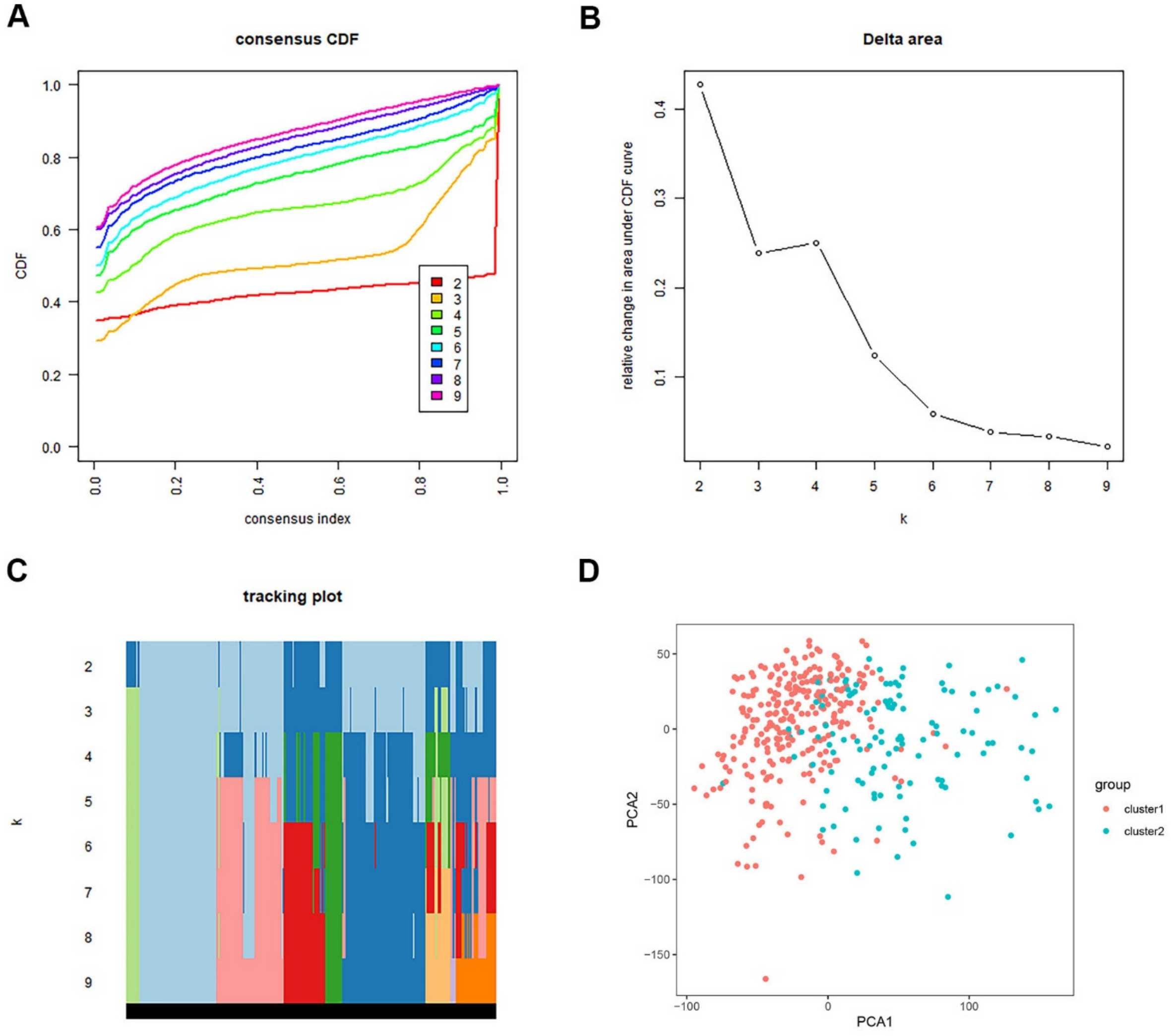

Figure S1. (A-C), consensus CDF, relative change in area under the CDF curve, and tracking plot for k=2 to 9. (D), PCA plot of mRNA expression profiles in HCC.

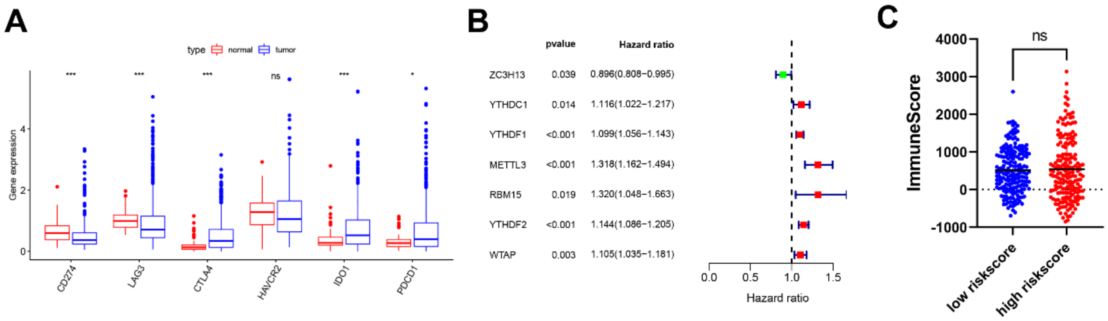

Figure S2. (A), the expression level of immune checkpoints between HCC and normal samples. (B), the forest plot of seven prognostic m6A genes filtered out by univariate Cox regression. (C), the relationship between risk score and immune score.

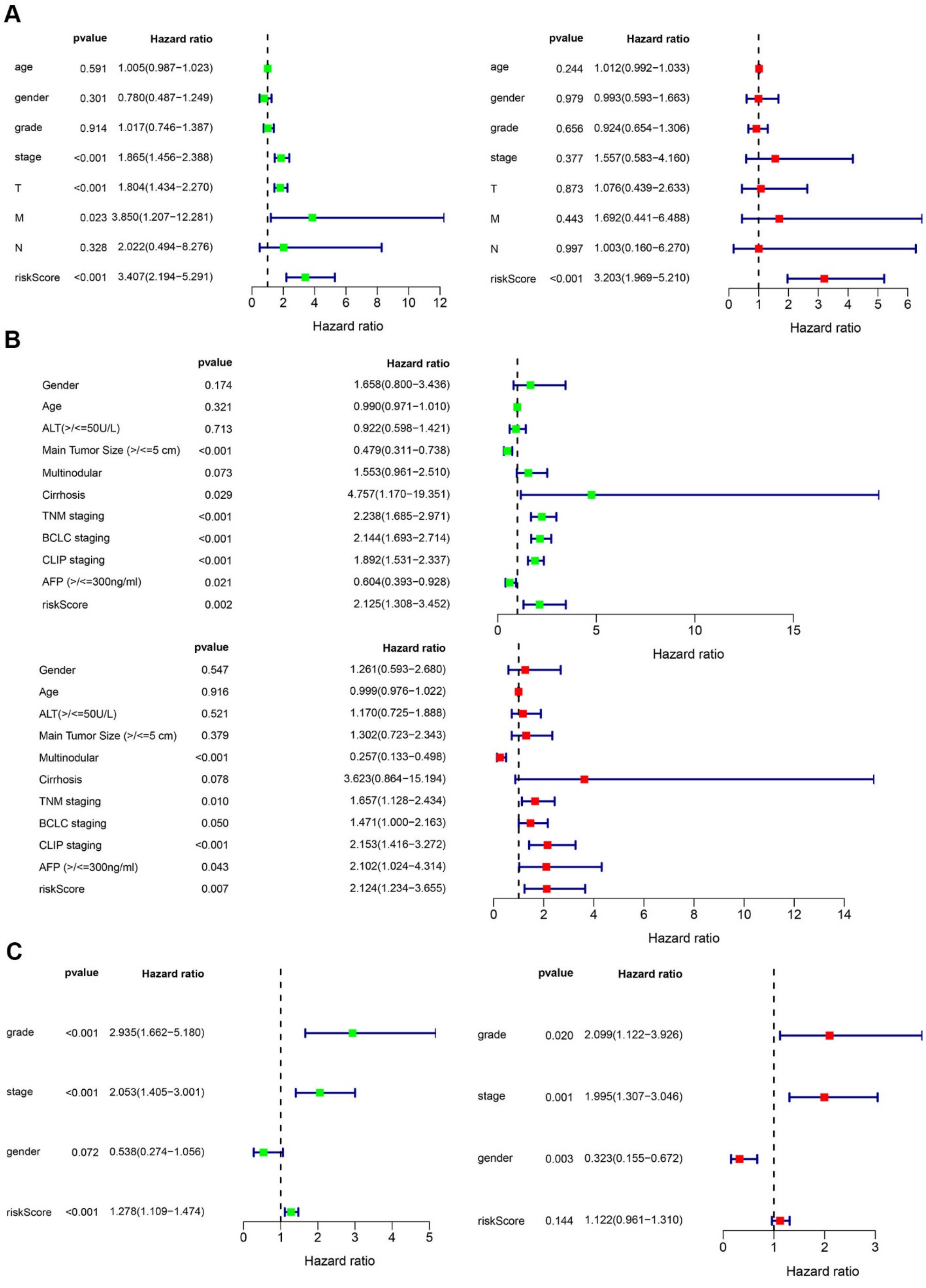

A
pvalue
Hazard ratio
age 0.591 1.005(0.987−1.023)
gender 0.301 0.780(0.487−1.249)
grade 0.914 1.017(0.746−1.387)
stage <0.001 1.865(1.456−2.388)
T <0.001 1.804(1.434−2.270)
M 0.023 3.850(1.207−12.281)
N 0.328 2.022(0.494−8.276)
riskScore <0.001 3.407(2.194−5.291)
Hazard ratio
pvalue
Hazard ratio
age 0.244 1.012(0.992−1.033)
gender 0.979 0.993(0.593−1.663)
grade 0.656 0.924(0.654−1.306)
stage 0.377 1.557(0.583−4.160)
T 0.873 1.076(0.439−2.633)
M 0.443 1.692(0.441−6.488)
N 0.997 1.003(0.160−6.270)
riskScore <0.001 3.203(1.969−5.210)
Hazard ratio
B
pvalue
Hazard ratio
Gender 0.174 1.658(0.800−3.436)
Age 0.321 0.990(0.971−1.010)
ALT(>/<=50U/L) 0.713 0.922(0.598−1.421)
Main Tumor Size (>/<=5 cm) <0.001 0.479(0.311−0.738)
Multinodular 0.073 1.553(0.961−2.510)
Cirrhosis 0.029 4.757(1.170−19.351)
TNM staging <0.001 2.238(1.685−2.971)
BCLC staging <0.001 2.144(1.693−2.714)
CLIP staging <0.001 1.892(1.531−2.337)
AFP (>/<=300ng/ml) 0.021 0.604(0.393−0.928)
riskScore 0.002 2.125(1.308−3.452)
Hazard ratio
pvalue
Hazard ratio
Gender 0.547 1.261(0.593−2.680)
Age 0.916 0.999(0.976−1.022)
ALT(>/<=50U/L) 0.521 1.170(0.725−1.888)
Main Tumor Size (>/<=5 cm) 0.379 1.302(0.723−2.343)
Multinodular <0.001 0.257(0.133−0.498)
Cirrhosis 0.078 3.623(0.864−15.194)
TNM staging 0.010 1.657(1.128−2.434)
BCLC staging 0.050 1.471(1.000−2.163)
CLIP staging <0.001 2.153(1.416−3.272)
AFP (>/<=300ng/ml) 0.043 2.102(1.024−4.314)
riskScore 0.007 2.124(1.234−3.655)
Hazard ratio
C
pvalue
Hazard ratio
grade <0.001 2.935(1.662−5.180)
stage <0.001 2.053(1.405−3.001)
gender 0.072 0.538(0.274−1.056)
riskScore <0.001 1.278(1.109−1.474)
Hazard ratio
pvalue
Hazard ratio
grade 0.020 2.099(1.122−3.926)
stage 0.001 1.995(1.307−3.046)
gender 0.003 0.323(0.155−0.672)
riskScore 0.144 1.122(0.961−1.310)
Hazard ratio

Figure S3. Univariate and multivariate Cox regression analyses in the TCGA cohort (A), the GEO cohort (B), and the ICGC cohort (C).

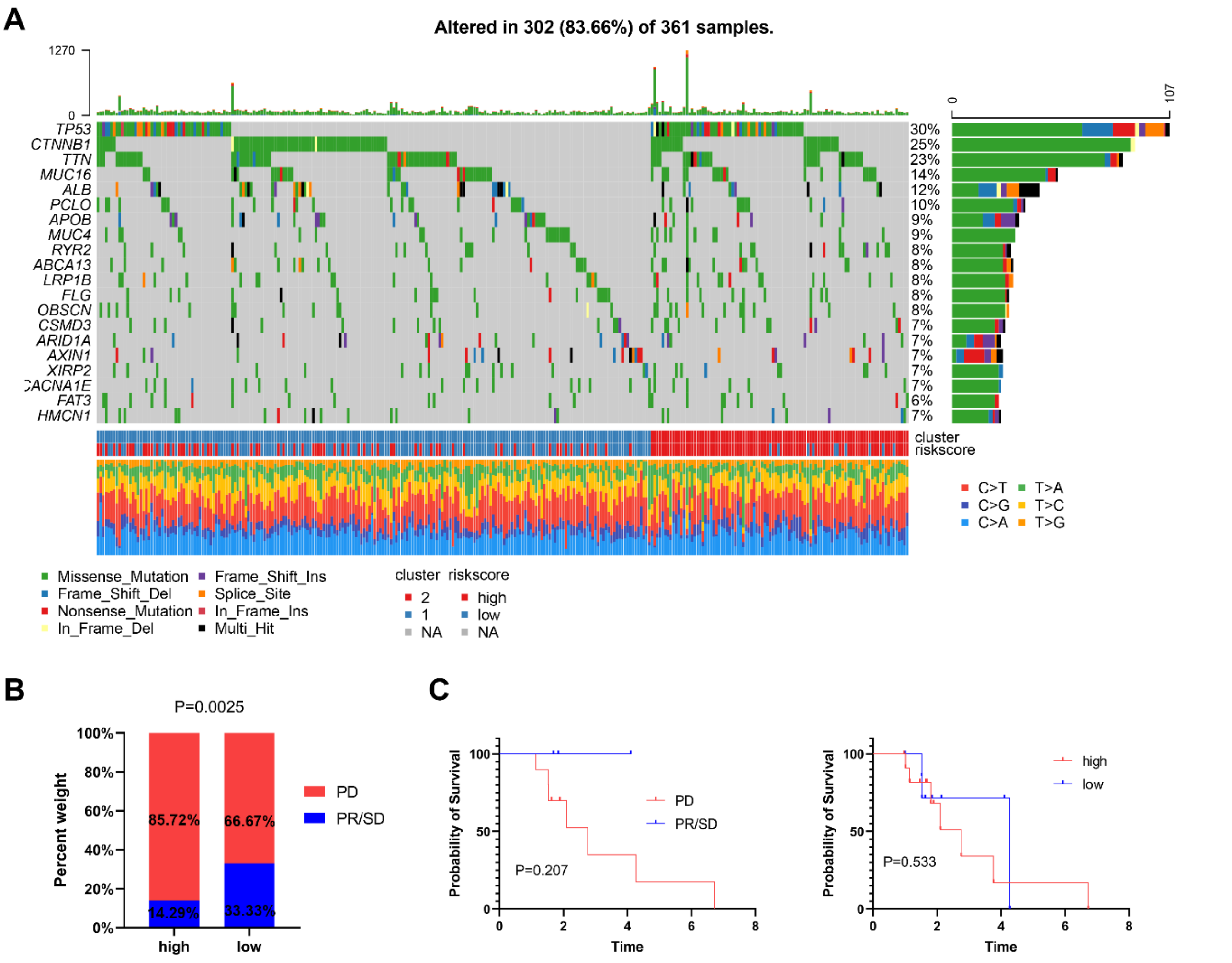

Figure S4. (A), the landscape of mutation in HCC stratified by cluster and risk signature. (B), Response to sorafenib treatment in the high-risk and low-risk patients in TCGA cohort. (C), Kaplan-Meier curve of the sorafenib-treated patients in TCGA cohort, stratified by response or risk score.
